# Supplementary material for: Cerium Oxides without U: The Role of Many-Electron Correlation
Source: J Phys Chem Lett. 2021 Jul 2;12(27):6277–83. doi: 10.1021/acs.jpclett.1c01589 (PMC8397342; doi:10.1021/acs.jpclett.1c01589)
Supplement: Supplementary file 1 — jz1c01589_si_001.pdf [file jz1c01589_si_001.pdf]

SUPPORTING INFORMATION FOR THE ARTICLE
Cerium Oxides without U: The Role of Many-Electron Correlation

Tobias Schäfer
Institute for Theoretical Physics, TU Wien, Wiedner Hauptstraße 8-10/136, 1040 Vienna, Austria

Nathan Daelman and Núria López
Institute of Chemical Research of Catalonia, The Barcelona Institute of Science and Technology, Tarragona, Spain

CONTENTS

I. General remarks on the computational settings and pseudopotentials 3

II. Ground states and metastable states of solid Ce_2O_3 3

III. The impact of the electronic structure on the U dependence 4

IV. Theory behind the correlation methods MP2, RPA, and RPA+rSOX 4

V. Basis set convergence and thermodynamic limit of the reaction energies 6

VI. Basis set convergence and thermodynamic limit of the lattice parameters 9

VII. Timings of correlation energy computations 10

VIII. Treatment of isolated molecules in a periodic code 11
1. HF, HSE, EXX 12
2. RPA and MP2 12

IX. Tables of reaction and atomization energies 13

X. Experimental references for the considered formation and reaction enthalpies 13

References 15

I. GENERAL REMARKS ON THE COMPUTATIONAL SETTINGS AND PSEUDOPOTENTIALS

All calculations are carried out with the Vienna *Ab Initio* Simulation Package (VASP) [29, 30]. The pseudopotentials employed are based on the frozen core approximation and the projector augmented wave method (PAW) [3], as specified in Table I. Those PAW potentials have been optimized for excited state properties (so-called GW POTCARs) including scalar relativistic effects. Furthermore, a norm-conserving (nc) PAW pseudopotential was used for Ce. Note that the pseudo-orbitals do not necessarily represent the charge of one electron, if non-norm-conserving PAW pseudopotentials are used, since the norm of the PAW pseudo-orbitals is not conserved in the core region. The calculation of expectation values thus requires so-called augmentation or compensation charges centered at the atoms. This correction is however missing for the unoccupied pseudo-orbitals, which leads to a poor description of unoccupied high-energy pseudo-orbitals. In turn, errors are introduced in correlation energies which strongly depend on the high-energy orbitals. The importance of norm-conserving PAW pseudopotentials for correlation calculations was previously recognized for 3d metals in Ref. [27]. For s, p bonded materials, the effect is usually negligible. While the impact for lattice constants of ceria is also negligible, the reaction enthalpies change dramatically, see Tab. II. In fact, if a non-norm conserving pseudpotential is used, about 2.5/2.9 eV of the correlation energy within the used method (MP2/RPA) is being missed for Ce_2O_3. For CeO_2 the effect is much smaller and of the order of -0.4/+0.3 eV, where the negative sign indicates an overcorrelation with non-norm conserving pseudpotentials.

Table I: List of explicitly treated valence electrons, core radii r_C, and default energy cutoffs `ENMAX` for the PAW potentials.

Element	Valence	r_C (Å)	`ENMAX` (eV)
H	$1s^1$	1.100	300.000
C	$2s^2 2p^2$	1.600	413.992
O	$2s^2 2p^4$	1.600	434.431
Ce (nc)	$5s^2 5p^6 6s^2 4f^1 5d^1$	2.400	630.493
Ce	$5s^2 5p^6 6s^2 4f^1 5d^1$	2.550	304.625

Table II: Comparison of the reaction enthalpy of r_1 and lattice constants between the norm conserving (nc) and non-norm-conserving pseudopotential of Ce. For Ce_2O_3 a fixed $c_0/a_0 = 1.5578$ was used.

Method	POTCAR	$\Delta_{r_1} H_{0\,\mathrm{K}}$	$a_0(CeO_2)$	$a_0(Ce_2O_3)$
MP2	Ce_GW_nc	3.2	5.366	3.870
MP2	Ce_GW	6.4	5.364	3.870
RPA	Ce_GW_nc	3.5	5.421	3.887
RPA	Ce_GW	5.8	5.415	3.885

The k-mesh is specified by three numbers ($k_1 \times k_2 \times k_3$), corresponding to a uniform sampling of the Brillouin zone in each direction of the reciprocal lattice, including the center ($\mathbf{\Gamma}$-point). Convergence of all calculated quantities with respect to the basis and k-mesh are discussed in sections V and VI.

Due to the presence of occupied f-orbitals, we include non-spherical one-center contributions to the gradient corrections (in GGA functionals like PBE or HSE03) inside the PAW spheres for all calculations. Additionally, the maximum angular momentum for augmenting the overlap densities in HF and correlation calculations is set to 6. The corresponding VASPflags are: `LASPH=TRUE`, `LMAXTAU=8`, `LMAXFOCK=6`, `LMAXFOCKAE=6`.

We performed spin-unrestricted open-shell calculations for the antiferromagnetic (AF) and ferromagnetic (FM) Ce_2O_3 crystal and the triplet O_2 molecule. The correct spin multiplet is enforced using the `NUPDOWN` flag. The correct symmetry for the FM and AF ground states of Ce_2O_3 is achieved by setting appropriate initial magnetic moments on the two Ce atoms in the primitive cell (`MAGMOM = 1.5 ±1.5 0.0 0.0 0.0`). As can be seen in Tab. III, the AF and FM ground states are virtually degenerate in each method considered. All presented results in the letter are based on AF ground states of Ce_2O_3. The impact of symmetry-broken antiferromagnetic supercells was not considered. Spin-restricted closed-shell calculations were carried out for solid CeO_2 and the molecules H_2, H_2O, CO_2, and CO.

II. GROUND STATES AND METASTABLE STATES OF SOLID Ce_2O_3

For open d- or f-shells materials, such as Ce_2O_3, self-consistent iterative algorithms (like conjugate gradient) are prone to getting stuck in local minima when applied to DFT+U, HF, or HSE functionals [9, 22, 32]. One physical

Table III: Difference of the ground state energies $\Delta H_{\mathrm{AF-FM}} = H_{\mathrm{AF}}^{\mathrm{Ce_2O_3}} - H_{\mathrm{FM}}^{\mathrm{Ce_2O_3}}$ of anti-ferromagnetic (AF) and ferromagnetic (FM) Ce_2O_3 as modeled by a single primitive cell.

Method	$\Delta H_{\mathrm{AF-FM}}$ / meV
HF	−0.7
MP2	+0.2
HSE03	−3.3
EXX@HSE03	+6.1
RPA@HSE03	−11.1

reason is the near degeneracy of the multiplet structure of the f-orbitals. Another technical reason is the convexity of the Fock exchange functional and the Hubbard U term with respect to the partial orbital occupancies [32]. Note that the Fock exchange and the Hubbard U are responsible for a sizeable band gap, while LDA and PBE predict a metallic behavior with partial occupancies close to the Fermi level.

To address this issue we make use of the ramping method by Meredig et al. [32] in order to find the correct semiconducting ground state at PBE + U and HSE03(α) level, based on the metallic ground state at the PBE level. In this method, the U or α parameter is gradually increased (starting from zero, i.e. PBE) while each self-consistent calculation starts with the orbitals and charge density from the previous calculation. In comparison to iterations starting from stochastic orbitals, we find that the ramping method converges to the lowest ground states in a robust and reproducible way.

III. THE IMPACT OF THE ELECTRONIC STRUCTURE ON THE U DEPENDENCE

The Mott-Hubbard Hamiltonian is designed to capture localization in strongly-correlated materials stemming from on-site Coulomb interaction. The literature suggests however that the 4f band in Ce_2O_3 is more complex in nature, presenting an unusually high degree of itinerancy.[12, 16, 17] Any such effects fall outside the on-site Hubbard correction and have to be captured by DFT. Alternatively, from the perspective of an (Anderson) impurity model itinerancy is best captured via hybridization with the surrounding Fermi liquid [13].

De Medici et al. has pointed out that both effects, i.e. localization and hybridization, are competitive in nature [8]. The authors demonstrated that at 0, K temperature the Mott transition is fully arrested due to the Kondo effect. In their Dynamic Mean-Field model any non-vanishing amount of hybridization suffices. This impediment is tempered at higher temperatures, where given a sufficiently high U value, the Mott transition can re-establish itself.

We propose that an analogous scenario transpires with DFT+U, due to the vanishing electron temperature. In the case of pure cerium there has been a better appreciation for the dual nature of Ce(4f) with an ongoing discussion into its impact on the γ-α phase transition [23]. The current consensus is that while the volume collapse may be explained by the Kondo effect at finite temperatures[18, 25, 42], Mott transitions still play a larger role at ultra-low temperatures [2, 6, 31]. The roles of lattice and electronic entropy herein are recognized as crucial [2, 31, 42].

IV. THEORY BEHIND THE CORRELATION METHODS MP2, RPA, AND RPA+RSOX

We express the electron-electron interaction energy E_{ee} in terms of mean-field spin-orbitals,

$$E_{\mathrm{ee}} = \frac{1}{2}\sum_{ij}^{\mathrm{occ}}[\langle ij|ij\rangle - \langle ij|ji\rangle] + \underbrace{\frac{1}{2}\sum_{ij}^{\mathrm{occ}}\sum_{ab}^{\mathrm{unocc}}\langle ij|ab\rangle[t_{ij}^{ab} - t_{ji}^{ab}]}_{\text{correlation energy } E_c} \tag{1}$$

where the composite indices $i, j, k, l, ... \in$ occ and $a, b, c, d, ... \in$ unocc label the occupied and unoccupied mean-field spin-orbitals, t_{ij}^{ab} are double excitation amplitudes and the electron-repulsion integrals read

$$\langle pq|rs\rangle = \int \mathrm{d}\boldsymbol{x} \int \mathrm{d}\boldsymbol{x}' \, \frac{\chi_p^*(\boldsymbol{x})\chi_r(\boldsymbol{x})\chi_q^*(\boldsymbol{x}')\chi_s(\boldsymbol{x}')}{|\boldsymbol{r}-\boldsymbol{r}'|} \;, \quad \chi_p(\boldsymbol{x}) = \varphi_p^\sigma(\boldsymbol{r}) \;, \sigma = \uparrow,\downarrow \tag{2}$$

The well-known MP2 approximation to the correlation energy E_c,

$$E_c^{\mathrm{MP2}} = \frac{1}{2}\sum_{ij}^{\mathrm{occ}}\sum_{ab}^{\mathrm{unocc}} \frac{\langle ij|ab\rangle[\langle ab|ij\rangle - \langle ab|ji\rangle]}{\varepsilon_i + \varepsilon_j - \varepsilon_a - \varepsilon_b} \;, \tag{3}$$

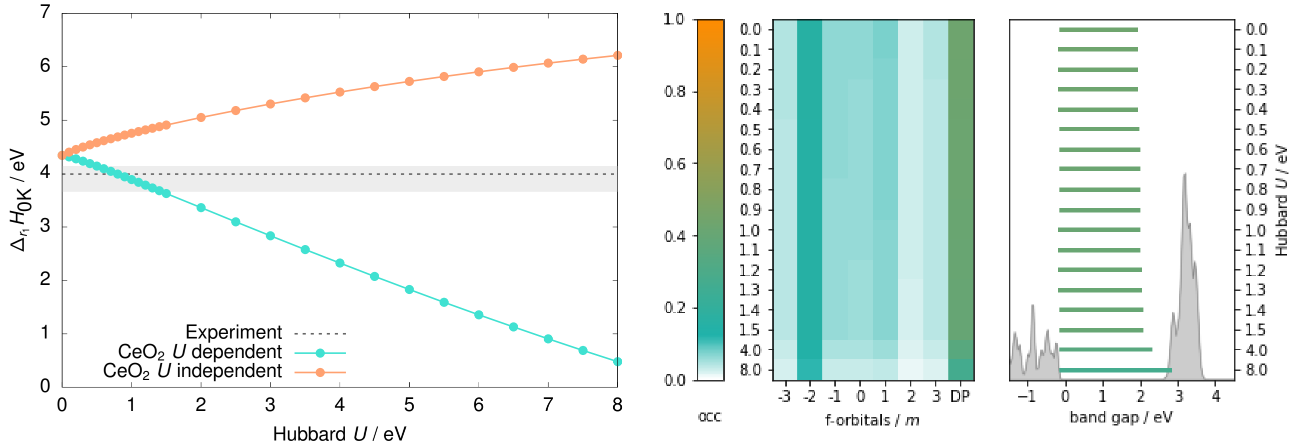

(a) Reaction enthalpy, $\Delta_{r_1}H_{0\,\mathrm{K}}$, for varying U

(b) CeO_2 electronic structure at PBE + U

Figure 1: (1a) Dependence on U for reaction enthalpy, $\Delta_{r_1}H_{0\,\mathrm{K}}$, under two scenarios. In the first option (green) accounts for a CeO_2 thermodynamic contribution that diminishes with increasing U. This scenario runs in parallel with the occupations shown in Figure 1b and recovers the trend shown in the main text's Figure 3 and Refs. [7] and [10]. The alternative (orange) assumes a fixed (PBE) energy term for CeO_2, as is typically observed in pristine slab systems [5]. The experimental reference value is denoted by the dashed, black line and its uncertainty in the grey zone. (1b) Parameter dependency of the f-band in bulk CeO_2 following the setup in Figure 2 of the main text. Similar observations were made in Refs. [7, 44].

corresponds to amplitudes defined by $t_{ij}^{ab} = \langle ab|ij\rangle/(\varepsilon_i+\varepsilon_j+\varepsilon_a-\varepsilon_b)$, where Hartree-Fock spin-orbitals χ and energies ε are used. The RPA correlation energy is computed by means of the independent-electron response function $P_0(\mathrm{i}\nu)$,

$$E_c^{\mathrm{RPA}} = \frac{1}{2}\int_0^\infty \frac{\mathrm{d}\nu}{2\pi}\,\mathrm{Tr}\left[\,\ln(\mathbb{1}-P_0(\mathrm{i}\nu)v_C)+P_0(\mathrm{i}\nu)v_C\right]\,, \tag{4}$$

with the Coulomb kernel $\langle \boldsymbol{r}|v_C|\boldsymbol{r}'\rangle = |\boldsymbol{r}-\boldsymbol{r}'|^{-1}$ and

$$\langle \boldsymbol{r}|P_0(\mathrm{i}\nu)|\boldsymbol{r}'\rangle = \sum_{\sigma=\uparrow,\downarrow}\sum_i^{\mathrm{occ}}\sum_a^{\mathrm{unocc}} \frac{\varphi_i^{\sigma*}(\boldsymbol{r})\varphi_i^{\sigma}(\boldsymbol{r}')\varphi_a^{\sigma*}(\boldsymbol{r}')\varphi_a^{\sigma}(\boldsymbol{r})}{\varepsilon_i^\sigma-\varepsilon_a^\sigma-\mathrm{i}\nu} + \mathrm{c.c.}\,. \tag{5}$$

As a basis Kohn-Sham spin-orbitals χ and energies ε from the HSE03 functional are used. In terms of the notation using amplitudes t_{ij}^{ab}, the RPA correlation energy corresponds to the neglect of the exchange-like term, i.e. $t_{ij}^{ab}-t_{ji}^{ab}\to t_{ij}^{ab}$ with amplitudes implicitly defined by

$$t_{ij}^{ab} = \Big[\,\langle ab|ij\rangle + \langle ak|ic\rangle t_{kj}^{cb} + t_{ik}^{ac}\langle kb|cj\rangle + t_{ik}^{ac}\langle kl|cd\rangle t_{lj}^{db}\,\Big]\;/\;(\varepsilon_i-\varepsilon_j+\varepsilon_a-\varepsilon_b)\,,$$

where a sum over only $k,l\in\mathrm{occ}$ and $c,d\in\mathrm{unocc}$ is understood. This can be interpreted as a simplification of the amplitude equation for coupled-cluster singles and doubles, restricting to the direct particle-hole ring-like diagrams [38]. The solution of (6) can analytically be expressed as an infinite sum over all ring-like Goldstone diagrams,

$$t_{ij}^{ab} = \frac{\langle ab|ij\rangle}{\varepsilon_i+\varepsilon_j-\varepsilon_a-\varepsilon_b} + 2\sum_k^{\mathrm{occ}}\sum_c^{\mathrm{unocc}}\frac{\langle bk|cj\rangle\langle ac|ik\rangle}{(\varepsilon_i+\varepsilon_k+\varepsilon_a-\varepsilon_b)(\varepsilon_i+\varepsilon_j-\varepsilon_a-\varepsilon_b)} + \dots\,. \tag{6}$$

The connection to Goldstone diagrams can be found in Figure 2 and Refs. [34, 41]. The inclusion of $-t_{ji}^{ab}$ is commonly denoted as RPA+SOSEX (RPA+second-order screened exchange), however, low-scaling algorithms are not yet available but possible [36]. Instead, we correct the missing exchange-like correlation in the RPA with a renormalized second-order approximation

$$-\,t_{ji}^{ab} = -\frac{\langle ab|ji\rangle}{f_{jj}+f_{ii}-f_{aa}-f_{bb}}\,, \tag{7}$$

where $f_{ij} = \langle i|f|j\rangle$ are matrix elements of the Fock matrix f in the Kohn-Sham spin-orbital basis. The denominator corresponds to a renormalization, $\varepsilon_j + \varepsilon_i - \varepsilon_a - \varepsilon_b - \Delta^{ab}_{ji}$ with $\Delta^{ab}_{ji} = (\varepsilon_j - f_{jj}) + (\varepsilon_i - f_{ii}) - (\varepsilon_a - f_{aa}) - (\varepsilon_b - f_{bb})$, where the ε's are the Kohn sham orbital energies. This renormalization is motivated by Epstein-Nesbet perturbation theory on top of the Kohn-Sham Hamiltonian [20]. It can be interpreted as an infinite resummation of (here) second-order exchange-like diagrams, as depicted in Figure 4 in Ref. [21]. In fact, the bare SOX contribution (i.e. $\Delta^{ab}_{ji} = 0$) suffers from the underestimated band gap of the Kohn-Sham basis. This would lead to a vast overcorrection of the exchange-like correlation, which is corrected by the renormalization Δ^{ab}_{ji} in rSOX.

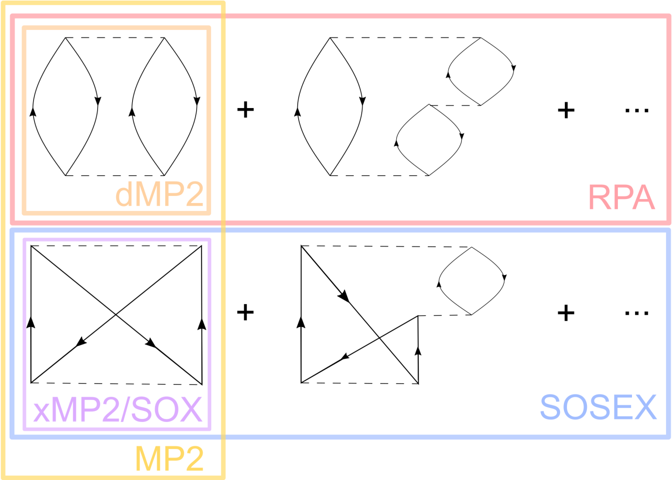

Figure 2: Goldstone diagrammatic representation of the various contributions to the correlation energy. The upper row consists solely of direct particle-hole ring-like diagrams, which correspond to RPA. Meanwhile, the lower row contains the exchange-like correlation, SOSEX. MP2 envelops the first-order diagrams of both rows in the HF basis. The SOX correction to RPA accounts for the first-order exchange-like contribution in the DFT basis. For the renormalized diagram, rSOX, we refer the reader to Figure 4 of Ref. [20].

V. BASIS SET CONVERGENCE AND THERMODYNAMIC LIMIT OF THE REACTION ENERGIES

Since we are using a periodic code, the reaction enthalpies primarily depend on two parameters, the basis set size (plane-wave cutoff `ENCUT`) and the k-mesh to sample the Brillouin zone (BZ). Considering the high computational cost of correlation energy calculations, a deliberate and efficient convergence scheme has to be considered in order to reach the complete basis set and thermodynamic limits.

All presented enthalpies correspond to zero-temperature and neglect the pV term. Hence, the reaction enthalpies ΔH are simply given by Helmholtz free energy differences, e.g. for the reduction $2CeO_2 + H_2 \rightarrow Ce_2O_3 + H_2O$,

$$\Delta H = H^{\mathrm{Ce_2O_3(s)}} - 2H^{\mathrm{CeO_2(s)}} + H^{\mathrm{H_2O(g)}} - H^{\mathrm{H_2(g)}} \ . \tag{8}$$

Here F denotes the Helmholtz free energy at $T = 0$, while (s) and (g) indicate the bulk and gas phase, respectively. Since the pV term is neglected, (g) corresponds to an isolated molecule. In Sec. VIII we present a scheme to calculate ground state energies of isolated molecules using the periodic VASP code.

Since both, periodic (solids) and aperiodic (isolated molecules) systems are involved, we apply the following scheme in order to find converged reactions energies.

1. Converge only the periodic part ($F^{\mathrm{Ce_2O_3(s)}} - 2F^{\mathrm{CeO_2(s)}}$) with respect to the number of k-points using a reasonable basis set size (`ENCUT` = 631 eV). Results can be found in Tab. IV, V, and VI for the HSE03, EXX+RPAc, and HF+MP2c levels of theory, respectively.

2. Converge the reaction energy ΔH (i.e. periodic and molecular part) with respect to the basis set size employing the previously found k-mesh for the periodic part. For the molecular part, use a relatively small cell size (about $L = 6.5$ or 5.0 Å for mean field or correlation energies, respectively) and a $2 \times 2 \times 2$ k-mesh (see Sec. VIII for justification). Results can be found in Tab. X, IX, and XI for the HSE03, EXX/RPAc, and HF/MP2c level of theory, respectively.

3. Extrapolate the cell size of the molecule to the case of an isolated molecule (as described in Sec. VIII) using the previously found basis set size and correct the molecular part of the energy from the previous step. Results are incorporated in the Tables X, IX, and XI, denoted as $\Delta H(L \to \infty)$.

The correlation methods employ an additional auxiliary plane-wave cutoff (`ENCUTGW` in VASP) for the calculation of two-electron repulsion integrals. It is first restricted to `ENCUTGW` $= 2/3$ `ENCUT`. In a follow-up step, both RPAc and MP2c extrapolate the internal auxiliary basis set, `ENCUTGW` $\to \infty$ [24, 27, 37]. This way the impact of `ENCUT` on the correlation energy is mainly left to unconverged mean-field orbitals. Note that the internal cutoff extrapolation is not used in the first step. The absolute correlation energies of step 1 and 2 must thus not be compared.

The referred tables allow for a robust estimation of the numerical error due to the finite k-meshes and basis set sizes. The error of the box size extrapolation for obtaining isolated molecules is estimated via the quality of the fit, see for instance Figure 4 and 7.

Table IV: k-point convergence of the periodic part at HSE03 level of theory using `ENCUT` $= 631\,\mathrm{eV}$.

Ce_2O_3 (AF)		CeO_2		$Ce_2O_3 - 2CeO_2$
k-points	F^{HSE03}	k-points	F^{HSE03}	$\Delta F^{\mathrm{HSE03}}$
$2\times2\times1$	−52.141	$2\times2\times2$	−31.333	10.525
$3\times3\times2$	−53.100	$3\times3\times3$	−31.534	9.967
$4\times4\times2$	−53.095	$4\times4\times4$	−31.534	9.973
$5\times5\times3$	−53.103	$5\times5\times5$	−31.534	9.966
$6\times6\times4$	−53.103	$6\times6\times6$	−31.534	**9.966**

Table V: k-point convergence of the periodic part at EXX@HSE03 and RPAc@HSE03 level of theory using `ENCUT` $= 631\,\mathrm{eV}$.

Ce_2O_3 (AF)			CeO_2			$Ce_2O_3 - 2CeO_2$
k-points	F^{EXX}	F^{RPAc}	k-points	F^{EXX}	F^{RPAc}	$\Delta F^{\mathrm{EXX+RPAc}}$
$2\times2\times1$	−94.939	−64.433	$2\times2\times2$	−54.721	−38.628	27.325
$3\times3\times2$	−96.101	−64.006	$3\times3\times3$	−55.018	−38.908	27.745
$4\times4\times2$	−96.123	−64.087	$4\times4\times4$	−55.001	−39.030	27.851
$5\times5\times3$	−96.073	−64.168	$5\times5\times5$	−55.000	−39.060	**27.819**
$6\times6\times4$	−96.071		$6\times6\times6$	−54.999		

Table VI: k-point convergence of the periodic part at HF and MP2 level of theory using `ENCUT` $= 631\,\mathrm{eV}$.

Ce_2O_3 (AF)			CeO_2			$Ce_2O_3 - 2CeO_2$	
k-points	F^{HF}	F^{MP2c}	k-points	F^{HF}	F^{MP2c}	ΔF^{HF}	ΔF^{MP2c}
$2\times2\times1$	−98.767	−44.631	$2\times2\times2$	−58.354	−26.061	17.942	7.491
$3\times3\times2$	−99.997	−45.472	$3\times3\times3$	−58.620	−26.479	17.244	7.487
$4\times4\times2$	−100.014	−45.556	$4\times4\times4$	−58.616	−26.563	17.218	**7.569**
$5\times5\times3$	−99.988	−45.609	$5\times5\times5$	−58.615	−26.593	17.243	7.576
$6\times6\times4$	−99.987		$6\times6\times6$	−58.615		**17.243**	

Table VII: Basis set convergence of the reaction enthalpy r_1 at HSE03 level. A k-mesh of $6 \times 6 \times 4$, $6 \times 6 \times 6$, and $2 \times 2 \times 2$ was employed for Ce_2O_3 (AF), CeO_2, and the molecule, respectively. The linear dimension of the cells containing the molecule is denoted by L.

ENCUT	Ce_2O_3	CeO_2	O_2	$\Delta H(L = 8.2\,\text{Å})$	$\Delta H(L \to \infty)$
631	−53.103	−31.534	−13.708	3.112	
680	−53.122	−31.539	−13.709	3.101	
730	−53.132	−31.540	−13.711	3.094	
780	−53.133	−31.540	−13.713	3.090	
830	−53.132	−31.539	−13.715	3.088	**3.088**

Table VIII: Basis set convergence of the reaction enthalpy r_1 at EXX@HSE03 level. A k-mesh of $6 \times 6 \times 4$, $6 \times 6 \times 6$, and $2 \times 2 \times 2$ was employed for Ce_2O_3 (AF), CeO_2, and the molecule, respectively. The linear dimension of the cells containing the molecule is denoted by L.

ENCUT	Ce_2O_3	CeO_2	O_2	$\Delta H(L = 8.2\,\text{Å})$	$\Delta H(L \to \infty)$
631	−96.071	−55.001	−29.987	−1.064	
680	−96.093	−55.001	−29.985	−1.082	
730	−96.095	−54.998	−29.981	−1.089	
780	−96.084	−54.992	−29.979	−1.089	
830	−96.072	−54.986	−29.977	−1.089	**−1.089**

Table IX: Basis set convergence of the reaction enthalpy r_1 at RPAc level. A k-mesh of $5 \times 5 \times 3$, $5 \times 5 \times 5$, and $2 \times 2 \times 2$ was employed for Ce_2O_3 (AF), CeO_2, and the molecule, respectively. The linear dimension of the cells containing the molecule is denoted by L.

ENCUT	Ce_2O_3	CeO_2	O_2	$\Delta H(L = 5.2\,\text{Å})$	$\Delta H(L \to \infty)$
631	−67.206	−40.856	−19.352	4.831	
680	−67.305	−40.877	−19.357	4.771	
730	−67.373	−40.888	−19.363	4.721	
780	−67.450	−40.911	−19.375	4.685	
830	−67.526	−40.921	−19.390	4.621	**4.614**
880	−67.560	−40.942	−19.399	4.624	
930	−67.582	−40.953	−19.404	4.622	

Table X: Basis set convergence of the reaction enthalpy r_1 at HF level. A k-mesh of $6 \times 6 \times 4$, $6 \times 6 \times 6$, and $2 \times 2 \times 2$ was employed for Ce_2O_3 (AF), CeO_2, and the molecule, respectively. The linear dimension of the cells containing the molecule is denoted by L.

ENCUT	Ce_2O_3	CeO_2	O_2	$\Delta H(L = 8.2\,\text{Å})$	$\Delta H(L \to \infty)$
631	−99.987	−58.615	−30.327	2.080	
680	−100.019	−58.624	−30.324	2.068	
730	−100.038	−58.634	−30.322	2.069	
780	−100.042	−58.640	−30.320	2.077	
830	−100.041	−58.642	−30.319	2.083	**2.083**

Table XI: Basis set convergence of the reaction enthalpy r_1 at MP2c level. A k-mesh of $4 \times 4 \times 2$, $4 \times 4 \times 4$, and $2 \times 2 \times 2$ was employed for Ce_2O_3 (AF), CeO_2, and the molecule, respectively. The linear dimension of the cells containing the molecule is denoted by L.

ENCUT	Ce_2O_3	CeO_2	O_2	$\Delta H(L = 5.2\,\text{Å})$	$\Delta H(L \to \infty)$
631	−46.230	−26.948	−12.977	1.178	
680	−46.219	−26.934	−12.968	1.165	
730	−46.240	−26.941	−12.972	1.156	
780	−46.272	−26.949	−12.987	1.132	
830	−46.314	−26.969	−13.006	1.121	**1.116**

VI. BASIS SET CONVERGENCE AND THERMODYNAMIC LIMIT OF THE LATTICE PARAMETERS

For each level of theory, the equation of state for CeO_2 and Ce_2O_3 was calculated by a Birch-Murnaghan fit of seven data points around ±10 % of the equilibrium volume. The convergence of the lattice parameters with respect to the employed k-mesh (i.e. the thermodynamic limit) and the basis set size is shown in Tab. XII and XIII for CeO_2 and Ce_2O_3, respectively. For all one-electron methods, we choose ENCUT $= 730$ and $6 \times 6 \times 6/4$, while for the correlation methods we choose ENCUT $= 631$ and $5 \times 5 \times 5/3$ for RPA, and ENCUT $= 631$ and $4 \times 4 \times 4/2$ for MP2. Note that the c/a ratio of Ce_2O_3 was kept fix during the convergence procedure, hence the presented results in Tab. XIII are not to be confused with a lattice relaxation. Fully relaxed structures are presented in the main publication.

Table XII: k-mesh and basis set convergence of the lattice parameters of CeO_2 at each level of theory. We consider the equilibrium lattice constant a_0 (and the corresponding unit cell volume V_0) as well as the bulk modulus B_0. Converged results are highlighted.

CeO_2			
ENCUT/eV	k-points	a_0/Å	B_0/GPa
HSE03			
631	$2 \times 2 \times 2$	5.422	205
631	$3 \times 3 \times 3$	5.398	205
631	$4 \times 4 \times 4$	5.398	206
631	$5 \times 5 \times 5$	5.398	206
631	$6 \times 6 \times 6$	5.398	206
680	$6 \times 6 \times 6$	5.398	206
730	$6 \times 6 \times 6$	5.399	206
EXX@HSE03			
631	$2 \times 2 \times 2$	5.423	258
631	$3 \times 3 \times 3$	5.400	260
631	$4 \times 4 \times 4$	5.402	260
631	$5 \times 5 \times 5$	5.402	260
631	$6 \times 6 \times 6$	5.402	260
680	$6 \times 6 \times 6$	5.402	260
730	$6 \times 6 \times 6$	5.402	261
RPA (= RPAc + EXX@HSE03@ENCUT=730@666k)			
631	$2 \times 2 \times 2$	5.425	204
631	$3 \times 3 \times 3$	5.425	202
631	$4 \times 4 \times 4$	5.422	201
631	$5 \times 5 \times 5$	5.421	202
631	$6 \times 6 \times 6$	5.421	202
680	$5 \times 5 \times 5$	5.420	201
730	$5 \times 5 \times 5$	5.420	201
HF			
631	$2 \times 2 \times 2$	5.472	228
631	$3 \times 3 \times 3$	5.453	230
631	$4 \times 4 \times 4$	5.454	228
631	$5 \times 5 \times 5$	5.453	229
631	$6 \times 6 \times 6$	5.454	229
680	$6 \times 6 \times 6$	5.454	229
730	$6 \times 6 \times 6$	5.454	229
MP2 (= MP2c + HF@ENCUT=730@666k)			
631	$2 \times 2 \times 2$	5.385	232
631	$3 \times 3 \times 3$	5.369	228
631	$4 \times 4 \times 4$	5.366	227
631	$5 \times 5 \times 5$	5.365	228
680	$4 \times 4 \times 4$	5.366	226
730	$4 \times 4 \times 4$	5.366	233

Table XIII: k-mesh and basis set convergence of the lattice parameters of Ce_2O_3 at each level of theory. We consider the equilibrium lattice constant a_0 (and the corresponding unit cell volume V_0) as well as the bulk modulus B_0 at the experimental c/a ratio of 1.5578 (hence, the presented numbers can not be considered as a lattice relaxation and differ from those in the main publication). Converged basis sets and k-meshes are highlighted.

Ce_2O_3 (AF)			
ENCUT/eV	k-points	a_0/Å	B_0/GPa
	HSE03		
631	$2\times2\times1$	3.891	139
631	$3\times3\times2$	3.876	144
631	$4\times4\times2$	3.877	143
631	$5\times5\times3$	3.877	143
631	$6\times6\times4$	3.877	143
680	$6\times6\times4$	3.877	143
730	$6\times6\times4$	3.877	143
	EXX@HSE03		
631	$2\times2\times1$	3.964	151
631	$3\times3\times2$	3.957	147
631	$4\times4\times2$	3.957	147
631	$5\times5\times3$	3.958	148
631	$6\times6\times4$	3.958	148
680	$6\times6\times4$	3.958	149
730	$6\times6\times4$	3.958	148
RPA (= RPAc + EXX@HSE03@ENCUT=730@664k)			
631	$2\times2\times1$	3.895	135
631	$3\times3\times2$	3.890	145
631	$4\times4\times2$	3.889	145
631	$5\times5\times3$	3.887	145
631	$6\times6\times4$	3.886	145
680	$5\times5\times3$	3.887	145
730	$5\times5\times3$	3.887	145
	HF		
631	$2\times2\times1$	3.954	142
631	$3\times3\times2$	3.953	139
631	$4\times4\times2$	3.952	140
631	$5\times5\times3$	3.953	142
631	$6\times6\times4$	3.953	141
680	$6\times6\times4$	3.953	141
730	$6\times6\times4$	3.953	141
MP2 (= MP2c + HF@ENCUT=730@664k)			
631	$2\times2\times1$	3.885	141
631	$3\times3\times2$	3.871	144
631	$4\times4\times2$	3.870	146
680	$4\times4\times2$	3.871	145
730	$4\times4\times2$	3.871	147
631	$5\times5\times3$	3.870	145

VII. TIMINGS OF CORRELATION ENERGY COMPUTATIONS

In Tab. XIV we present the computation time for the correlation methods RPA and MP2 as well as the rSOX method for RPA+rSOX. For a clearer ranking, we also specify the basis set size by the plane-wave cutoff ENCUT and the number of unoccupied orbitals, as well as the number of k-points in the full and in the irreducible Brillouine zone (BZ). The calculations were performed on Intel Xeon Platinum 8160 CPUs.

Table XIV: Timings for the different correlation methods and phases of ceria. The number of unoccupied orbitals (#unocc.) per k-point is rounded to hundreds, since VASP generates slightly different plane-wave meshes (basis set sizes) for each k-point.

method	phase	ENCUT/eV	#unocc.	k-points	k-points (irr. BZ)	cores	time / hours
RPA	CeO_2	830	2200	125	10	128	0.6
rSOX	CeO_2	830	2200	27	4	128	5.7
MP2	CeO_2	830	2200	64	8	1024	8.5
RPA	Ce_2O_3	830	4300	75	12	128	1.4
rSOX	Ce_2O_3	830	4300	9	6	512	25.6
MP2	Ce_2O_3	830	4300	32	8	2048	20.8

VIII. TREATMENT OF ISOLATED MOLECULES IN A PERIODIC CODE

For the reactions, we aim to find the ground state energy (equivalent to the Helmholtz free energy at $T = 0$) of the isolated molecules H_2O, H_2, CO_2, CO, and O_2. In a periodic code, an isolated molecule can be simulated either by using a sufficiently large unit cell or by explicitly targeting the limiting case of an infinitely dilute gas by means of predictive cell size extrapolations. In the first case, periodicity is troublesome and the distance between periodic images has to be sufficiently large. In the second approach, periodicity is incorporated by interpreting the simulation as a gas of molecules (note, that ”gas” is understood only as a technical term for periodically repeated molecules). Here, we chose the second approach for the following reason: the correlation energy varies inversely with the HOMO-LUMO gap of the mean-field solution, while the HOMO-LUMO gap itself converges only slowly with the cell size. Hence, undesirable large unit cells have to be employed in order to achieve a converged correlation energy of an isolated molecule. Contrariwise, a predictive cell size extrapolation allows to restrict to smaller cells by employing fitting functions, which cover the physical long-range interactions between molecules and the effect of the HOMO-LUMO gap.

In order to simulate a gas of molecules in a periodic code, the following technical aspects have to be considered. The number of interacting molecules is determined by the number of molecules in the Born-von Karman (BvK) cell. With computational efficiency in mind, we increase this number by a uniform $n_k \times n_k \times n_k$ sampling of the Brillouine zone, being equivalent to a supercell containg $N_k = n_k^3$ molecules. The volume of the BvK cell is then given by $\Omega = N_k\Omega_0$, while Ω_0 is the volume of the unit cell containting only one molecule. The use of $n_k = 1$ would prohibit any physical meaningful interaction between molecules, hence $n_k \geq 2$ is required. This can be seen by the construction of the electron-electron Coulomb interaction in reciprocal space: $\widetilde{v}(\boldsymbol{G}) = 4\pi/\boldsymbol{G}^2$ with $\boldsymbol{G} = \boldsymbol{g} + \boldsymbol{k}$ where $\boldsymbol{g}$ is a reciprocal lattice vector and $\boldsymbol{k}$ is a sampling vector of the Brillouin zone (BZ). In real space the Coulomb kernel corresponds to the discrete Fourier series of $\widetilde{v}$,

$$v(\boldsymbol{r}-\boldsymbol{r}') = \frac{1}{N_k}\sum_{\boldsymbol{k}}^{\mathrm{BZ}}\sum_{\boldsymbol{g}} \widetilde{v}(\boldsymbol{g}+\boldsymbol{k})\, \mathrm{e}^{\mathrm{i}(\boldsymbol{g}+\boldsymbol{k})(\boldsymbol{r}-\boldsymbol{r}')} \; . \tag{9}$$

Equation (9) is equivalent to $1/|\boldsymbol{r}-\boldsymbol{r}'|$ only in the thermodynamic limit, i.e. $N_k \to \infty$ and $\frac{1}{N_k}\sum_{\boldsymbol{k}}^{\mathrm{BZ}} \to \int_{\mathrm{BZ}} \frac{\mathrm{d}^3k}{(2\pi)^3}$. The deviation is particularly substantial in the case of a $\boldsymbol{\Gamma}$-only sampling of the BZ, i.e. $N_k = n_k = 1$ and thus $\frac{1}{N_k}\sum_{\boldsymbol{k}}^{\mathrm{BZ}} \to 1$:

$$v(\boldsymbol{r}-\boldsymbol{r}'+\boldsymbol{R}) = v(\boldsymbol{r}-\boldsymbol{r}') \; . \tag{10}$$

The Coulomb potential in real space is then periodic along each lattice vector $\boldsymbol{R}$ and $v(\boldsymbol{r}-\boldsymbol{r}')$ takes only intramolecular interaction into account, while intermolecular interactions (with distances of the order of multiples of $\boldsymbol{R}$) are mapped back to intramolecular interactions. In an intermediate regime, where the unit cell does not substantially exceed the spatial extend of the electron density of the molecule, the periodicity of $v(\boldsymbol{r}-\boldsymbol{r}')$ leads to a spurious mixing of intramolecular and intermolecular interaction. In this case, the BvK boundary conditions are simply unphysical.

In passing we note that this argument is not valid for extended molecules, where significant intermolecular interactions can be of much smaller distance compared to the linear dimension of the BvK cell. A $\boldsymbol{\Gamma}$-only approach can then still be sufficient though. Since we are considering only small molecules, our approach of a predictive extrapolation of the intermolecular interactions requires a denser k-point sampling. This is particularly important to capture the dispersion interactions in correlation methods and will be discussed in the following subsections.

A further challenge arises from the $\boldsymbol{g}+\boldsymbol{k}=0$ contribution in Eq. (9), which corresponds to the long-range interaction in real space, i.e. $|\boldsymbol{r}-\boldsymbol{r}'| \to \infty$. Although integrable in the thermodynamic limit, any finite k-mesh requires a special context-dependent treatment to avoid the numerical divergence. We discuss the solutions to this problem in the relevant subsections below.

1. *HF, HSE, EXX*

While the convergence of periodic DFT calculations of a molecule with respect to the linear unit cell dimension $L = \Omega_0^{1/3}$ is rather cheap, the introduction of the Fock operator (as in HF or hybrid functional calculations) poses a particular challenge. This is due to the fact, that the Fock exchange operator suffers from unphysical electrostatic interactions between BvK cells, when assuming periodic boundary conditions with a finite k-mesh (i.e. a finite volume of the BvK cell) [14]. Since the leading contributions of those interactions are of monopole-monopole character, the Fock exchange energy is divergent for any finite k-mesh. Formally, in a periodic code, the divergence of the exchange energy becomes apparent by the $\boldsymbol{g}+\boldsymbol{k}=0$ contribution in Eq. (9). Note that the difference between the classical electrostatic interaction (Hartree term) and the exchange interaction is that the former is largely compensated for by the positively charged nuclei, thereby preventing divergence.

Correcting the divergence from the exchange energy is thus mandatory. Among other known approaches [40], we consider two strategies to address this issue. (i) The first is the probe-charge method (default in VASP) [33], which corrects for the spurious monopole-monopole interactions between BvK cells. This leaves us with non-diverging monopole-dipole interactions that are sill unphysical however. (ii) The second one is the Alavi-Spencer scheme [39], which applies a simple radial cutoff to the Coulomb interaction in the Fock operator (`HFRCUT=-1` in VASP). It essentially crops the interactions between BvK cells.

For range-separated functionals like the HSE03 hybrid functional, the situation is less demanding. By definition the Fock operator is restricted here to short-ranged interactions. These are enforced by multiplying the Coulomb interaction with the complementary error function, $\mathrm{erfc}(0.3 \cdot |\boldsymbol{r}-\boldsymbol{r}'|)$, which suppresses the interactions beyond 6.67 Å. The long-ranged exchange interactions are left to the quicker decaying PBE exchange functional. The exact exchange energy (EXX, i.e. the Hartree-Fock functional evaluated using HSE03 orbitals) is again exposed to the same divergence problem described above however and requires correction.

Technically, the physically meaningful range of the Coulomb interaction (10) is limited by the number of k-points in VASP, if the unit cell contains only one molecule. To accurately capture and extrapolate the electrostatic long-ranged electrostatic interactions, a k-point sampling beyond the $\boldsymbol{\Gamma}$-only approach is necessary. This constitutes the first step in our practical scheme for finding the limit of an infinitely dilute gas (isolated molecule) at the HF, HSE03 and EXX level. (i) We increase the number of k-points until the ground state energy is converged to less than 1 meV, using a fixed but reasonable large L (usually about 6 Å). This step ensures that the ground state energies are free from unphysical exchange interactions between BvK cells and that enough molecular images are incorporated (measured by the number of k-points) to accurately capture the intermolecular interaction energy per molecule. (ii) We extrapolate the ground state energy using $E_0 + \alpha L^{-3} + \beta L^{-6}$, which accounts for intermolecular electrostatic monopole-dipole (L^{-3}) and dipole-dipole interactions (L^{-6}). We sample the data points over the range of $L = 6$ to 10 Å. The k-mesh has been determined in the previous step and remains fixed throughout.

In these systems we use symmetry breaking orthorhombic unit cells, however, the parameter $L = \Omega_0^{1/3}$ is still adopted as the linear cell dimension. Comparing approaches, the probe-charge method on the suffers from the slowly decaying unphysical monopole-dipole interactions between BvK cells in the exchange energy. The Alavi-Spencer scheme on the other hand converges exponentially with respect to the number of k-points [39]. So even a $2 \times 2 \times 2$ mesh is sufficient for all considered molecules if $L \geq 6$ Å, as illustrated in the left plot of Figure 3. We therefore regard the Alavi-Spencer scheme as superior for our setup. Indeed, the intermolecular HSE03 energies all nicely converge for a $2 \times 2 \times 2$ k-mesh, as exemplified in the right plot in Figure 3. Similarly to HF, the EXX energy converges faster with the Alavi-Spencer scheme. Using this setting, the HF and HSE03 energy can then be extrapolated with an accuracy of 1 meV to the case of an isolated molecule (infinitely dilute gas), see left panel of Figure 4.

2. *RPA and MP2*

A more subtle task is to perform a cell size extrapolation of the correlation energy with predictive power to the limit of an isolated molecule. In the following, we derive a fitting function to extrapolate the correlation energy with respect to the linear unit cell dimension L, using the MP2 method as an example. The MP2 correlation energy of distant molecules with non-overlapping electron densities can be written as a sum over intramolecular and intermolecular pairs:

$$E^{\text{MP2c}} = E^{\text{MP2c}}_{\text{intra}} + E^{\text{MP2c}}_{\text{inter}} \; , \qquad E^{\text{MP2c}}_{\text{intra}} = \sum_{M}^{\text{molec.}} \sum_{ij\in M}^{\text{occ.}} e_{ij} \; , \qquad E^{\text{MP2c}}_{\text{inter}} = \sum_{M_1\neq M_2}^{\text{molec.}} \sum_{\substack{i\in M_1\\ j\in M_2}}^{\text{occ.}} e_{ij} \; , \tag{11}$$

$$e_{ij} = \sum_{ab}^{\text{unocc.}} \frac{v^{ij}_{ab}(2v^{ab}_{ij} - v^{ab}_{ji})}{\varepsilon_i + \varepsilon_j - \varepsilon_a - \varepsilon_b} \qquad v^{ij}_{ab} = \int \mathrm{d}^3 r \int \mathrm{d}^3 r' \; \varphi^*_i(\boldsymbol{r})\varphi_a(\boldsymbol{r}) v(\boldsymbol{r}-\boldsymbol{r}')\varphi^*_j(\boldsymbol{r}')\varphi_b(\boldsymbol{r}') \; . \tag{12}$$

The Coulomb integrals $2v^{ij}_{ab}v^{ab}_{ij}$ in the inter pairs account for the London dispersion interaction, L^{-6}. Meanwhile, the L dependency of the intra pairs is mainly driven by the change of the energetic distance between the occupied and unoccupied energies in the denominator, $(\varepsilon_i + \varepsilon_j - \varepsilon_a - \varepsilon_b)^{-1}$, as indicated by the HOMO-LUMO gap in Figure 5. We thus assume a behavior following $(1+\gamma L^{-3})^{-1} = 1 - \gamma L^{-3} + \mathcal{O}(L^{-6})$ for the intra pairs, since the gap closes largely with L^{-3}. Thus, for large L the slow convergence of the intramolecular correlation dominates over the more rapid convergence of the intermolecular correlation. A predictive extrapolation of the MP2 correlation energy should therefore cover both the closing HOMO-LUMO gap (L^{-3}) and the dispersion interaction (L^{-6}):

$$E_\infty + \alpha L^{-3} + \beta L^{-6} \; , \tag{13}$$

The L^{-3} term here is not to be confused with the monopole-dipole interaction in Section VIII 1. Actually, the densities $\varphi^*_i(\boldsymbol{r})\varphi_a(\boldsymbol{r})$ are monopole free in perturbation theory, since the occupied and unoccupied manifolds are mutually orthogonal. In the particular case of MP2, the exchange part $-v^{ij}_{ab}v^{ab}_{ji}$ is of very local nature and therefore contributes only to the intra pairs. Its opposite sign slightly mitigates the impact of the closing HOMO-LUMO gap, whereas RPA correlation energies lack exchange terms.

As discussed in the previous section, the range of the Coulomb interaction is technically limited by the number of k-points, if the unit cell contains only one molecule. Using a $\boldsymbol{\Gamma}$-only sampling of the BZ, the correlation energy is thus expected to show only the L^{-3} behavior of the closing HOMO-LUMO gap. A $2\times2\times2$ sampling of the BZ however can at least capture the dispersion interaction between nearest neighbors. Although the missing interaction of more distant neighbours decays only with N_k^{-1}, the extrapolation (13) is remarkably stable with respect to the k-meshes employed, see left plot in Figure 6. A denser k-mesh captures more intermolecular dispersion per molecule, thus increasing the ratio $|\beta/\alpha|$ in Eq. (13). This leads to a further ranged dominance of the L^{-6} term. A similar effect can be observed when a finite size correction (FS) for the $\boldsymbol{g}+\boldsymbol{k}=0$ contribution to the correlation energy is employed in Eq. (9). We apply FS using kp-perturbation theory [11], the output of which VASP stores into the `WAVEDER` file. Apparently, the missing long-ranged intermolecular dispersion is overcorrected again, leading to a further ranged dominance of the L^{-6} term.

The failure of the $\boldsymbol{\Gamma}$-only approach is evident from Figure 5. The HOMO-LUMO gap converges there under a constant slope, while finer k-meshes reach the final slope earlier. Thus the extrapolation (13) predicts a too small energy gap in the denominator for $\boldsymbol{\Gamma}$-only, which in turn yields too strong correlation.

We conclude that using a $2\times2\times2$ mesh and Eq. (13) suffices to extrapolate the MP2 correlation energy to the limit of an infinitely dilute gas for all molecules under consideration. The correction of the $\boldsymbol{g}+\boldsymbol{k}=0$ contribution (`WAVEDER`) does not lead to significantly more accurate extrapolations. Since our MP2 implementation that automatically extrapolates the basis set does not support the $\boldsymbol{g}+\boldsymbol{k}=0$ correction, we neglected this correction in favour of the basis set extrapolation. Finally, the cell size extrapolated results are supported by the MP2 calculations using a truncated Coulomb interaction (Figure 6). In passing we note that truncating the Coulomb kernel in correlation methods is meaningful only if the HOMO-LUMO gap of the underlying mean field solution is fully converged, hence very large L have to be considered.

Likewise, the RPA correlation energies are extrapolated using Eq. (13), since the arguments apply to all perturbative correlation energy methods. Figure 7 (right plot) presents extrapolated RPA correlation energies.

IX. TABLES OF REACTION AND ATOMIZATION ENERGIES

X. EXPERIMENTAL REFERENCES FOR THE CONSIDERED FORMATION AND REACTION ENTHALPIES

In this section we compile the experimental data used in our work. All energies are per formula unit. The computed Helmholtz free energies (F) are equivalent to enthalpies (H) at $T = 0\,\text{K}$ if the pV term is neglected, which

Table XV: Enthalpies of reaction and formation at zero temperature.

Method	$\Delta_{r_1} H_{0\,\mathrm{K}}$	$\Delta_{r_2} H_{0\,\mathrm{K}}$	$\Delta_{r_3} H_{0\,\mathrm{K}}$	$\Delta_\mathrm{f} H_{0\,\mathrm{K}}^{\mathrm{CeO_2}}$	$\Delta_\mathrm{f} H_{0\,\mathrm{K}}^{\mathrm{Ce_2O_3}}$	Refs.
**RPA**	3.52	0.99	0.66	−10.79	−18.10	
**RPA+rSOX**	3.97	1.19	0.91			
**MP2**	3.18	0.34	−0.10			
HF	2.07	−0.34	−0.31	−13.24	−24.41	
HSE03 ($\alpha = 0.25$)	3.09	0.46	−0.13	−11.02	−18.94	
PBE + U (4.5 eV)	2.29					[7]
PBE	4.18			−10.24	−16.30	
LDA	4.92			−11.49	−18.07	
**Expt. (selected)**	3.99	1.26	0.96	−11.32	−18.65	see Sec. X
**Expt. (range)**	3.7–4.1	1.0–1.4	0.7–1.1		(−18.9)–(−18.5)	see Sec. X

Table XVI: Zero-temperature atomization energies in eV per atom. The experimental values are ZPE corrected. For the solids this corresponds to the negative cohesive energy per atom.

Method	CeO_2(s)	Ce_2O_3(s)	α-Ce(s)	O_2(g)	CO_2(g)	CO (g)	H_2O (g)	H_2(g)	Refs.
**RPA**	6.66	6.80	4.26	2.46	5.35	5.37	3.22	2.33	
**MP2**	7.28	7.53		2.82	5.97	5.89	3.41	2.29	
HF	4.41	4.74	−1.44	0.71	3.57	3.79	2.25	1.82	
HSE03 ($\alpha = 0.25$)	6.55	6.71	3.25	2.70	5.67	5.55	3.29	2.27	
PBE + U (4.5 eV)									[7]
PBE				3.11					[7]
LDA				3.78					[7]
**Expt. (selected)**	6.96	7.03	4.34	2.61	5.63	5.62	3.36	2.37	see Sec. X
**Expt. (range)**		6.98–7.05							see Sec. X

is a reasonable assumption for solids and gases at standard pressure. Hence, F and $H_{0\,\mathrm{K}}$ are used as synonyms. Furthermore, for a better comparison with electronic *ab initio* free energies, we correct all experimental enthalpies for zero-point energies (ZPE) as listed in Tab. XVII.

We consider the reactions

$$2\,\mathrm{CeO_2(s)} \longrightarrow \mathrm{Ce_2O_3(s)} + \frac{1}{2}\,\mathrm{O_2(g)} \tag{r$_1$}$$

$$2\,\mathrm{CeO_2(s)} + \mathrm{H_2(g)} \longrightarrow \mathrm{Ce_2O_3(s)} + \mathrm{H_2O\,(g)} \tag{r$_2$}$$

$$2\,\mathrm{CeO_2(s)} + \mathrm{CO\,(g)} \longrightarrow \mathrm{Ce_2O_3(s)} + \mathrm{CO_2(g)} \tag{r$_3$}$$

and derive the experimental reaction enthalpies, $\Delta_r H_{0\,\mathrm{K}}$, from available experimental atomization enthalpies, $\Delta_\mathrm{at} H_{0\,\mathrm{K}}$,

$$\Delta_{r_1} H_{0\,\mathrm{K}} = \Delta_\mathrm{at} H_{0\,\mathrm{K}}^{\mathrm{Ce_2O_3(s)}} + \tfrac{1}{2}\Delta_\mathrm{at} H_{0\,\mathrm{K}}^{\mathrm{O_2(g)}} - 2\Delta_\mathrm{at} H_{0\,\mathrm{K}}^{\mathrm{CeO_2(s)}}\;, \tag{14}$$

$$\Delta_{r_2} H_{0\,\mathrm{K}} = \Delta_\mathrm{at} H_{0\,\mathrm{K}}^{\mathrm{Ce_2O_3(s)}} + \Delta_\mathrm{at} H_{0\,\mathrm{K}}^{\mathrm{H_2O\,(g)}} - 2\Delta_\mathrm{at} H_{0\,\mathrm{K}}^{\mathrm{CeO_2(s)}} - \Delta_\mathrm{at} H_{0\,\mathrm{K}}^{\mathrm{H_2(g)}}\;, \tag{15}$$

$$\Delta_{r_3} H_{0\,\mathrm{K}} = \Delta_\mathrm{at} H_{0\,\mathrm{K}}^{\mathrm{Ce_2O_3(s)}} + \Delta_\mathrm{at} H_{0\,\mathrm{K}}^{\mathrm{CO_2(g)}} - 2\Delta_\mathrm{at} H_{0\,\mathrm{K}}^{\mathrm{CeO_2(s)}} - \Delta_\mathrm{at} H_{0\,\mathrm{K}}^{\mathrm{CO\,(g)}}\;. \tag{16}$$

While zero-temperature atomization energies are available [1] for the gases (the second value is the ZPE correction),

$$\Delta_\mathrm{at} H_{0\,\mathrm{K}}^{\mathrm{H_2(g)}} = -4.478\,\mathrm{eV} - 0.269\,\mathrm{eV} = -4.747\,\mathrm{eV} \tag{17}$$

$$\Delta_\mathrm{at} H_{0\,\mathrm{K}}^{\mathrm{H_2O\,(g)}} = -9.512\,\mathrm{eV} - 0.574\,\mathrm{eV} = -10.086\,\mathrm{eV} \tag{18}$$

$$\Delta_\mathrm{at} H_{0\,\mathrm{K}}^{\mathrm{CO\,(g)}} = -11.108\,\mathrm{eV} - 0.134\,\mathrm{eV} = -11.242\,\mathrm{eV} \tag{19}$$

$$\Delta_\mathrm{at} H_{0\,\mathrm{K}}^{\mathrm{CO_2(g)}} = -16.562\,\mathrm{eV} - 0.314\,\mathrm{eV} = -16.876\,\mathrm{eV} \tag{20}$$

$$\Delta_\mathrm{at} H_{0\,\mathrm{K}}^{\mathrm{O_2(g)}} = -5.117\,\mathrm{eV} - 0.098\,\mathrm{eV} = -5.215\,\mathrm{eV} \tag{21}$$

we derive the atomization enthalpies for the bulk cerium oxides using

$$\Delta_\mathrm{at} H_{0\,\mathrm{K}}^{\mathrm{CeO_2(s)}} = \Delta_\mathrm{f} H_{0\,\mathrm{K}}^{\mathrm{CeO_2(s)}} + \Delta_\mathrm{at} H_{0\,\mathrm{K}}^{\alpha\text{-}\mathrm{Ce(s)}} + \Delta_\mathrm{at} H_{0\,\mathrm{K}}^{\mathrm{O_2(g)}}\;, \tag{22}$$

$$\Delta_\mathrm{at} H_{0\,\mathrm{K}}^{\mathrm{Ce_2O_3(s)}} = \Delta_\mathrm{f} H_{0\,\mathrm{K}}^{\mathrm{Ce_2O_3(s)}} + 2\Delta_\mathrm{at} H_{0\,\mathrm{K}}^{\alpha\text{-}\mathrm{Ce(s)}} + \frac{3}{2}\Delta_\mathrm{at} H_{0\,\mathrm{K}}^{\mathrm{O_2(g)}}\;. \tag{23}$$

The experimental atomization enthalpy of solid α-Ce can be found in Refs. [4, 26] and reads

$$\Delta_{\mathrm{at}} H_{0\,\mathrm{K}}^{\alpha\text{-Ce(s)}} = -4.32\,\mathrm{eV} - 0.02\,\mathrm{eV} = -4.34\,\mathrm{eV}\ . \tag{24}$$

For the experimental formation enthalpies of both cerium oxides only room temperature measurements are available in the literature [28]: $\Delta_{\mathrm{f}} H_{298.15\,\mathrm{K}}^{\mathrm{CeO_2(s)}} = -11.30\,\mathrm{eV}$ and $\Delta_{\mathrm{f}} H_{298.15\,\mathrm{K}}^{\mathrm{Ce_2O_3(s)}} = -18.65\,\mathrm{eV}$. We assume that finite temperature effects are small and estimate the zero-temperature formation enthalpies only by correcting for the ZPE,

$$\Delta_{\mathrm{f}} H_{0\,\mathrm{K}}^{\mathrm{CeO_2(s)}} = \Delta_{\mathrm{f}} H_{298.15\,\mathrm{K}}^{\mathrm{CeO_2(s)}} - \left(\mathrm{ZPE}^{\mathrm{CeO_2(s)}} - \mathrm{ZPE}^{\alpha\text{-Ce(s)}} - \mathrm{ZPE}^{\mathrm{O_2(g)}}\right) = -11.32\,\mathrm{eV}\ , \tag{25}$$

$$\Delta_{\mathrm{f}} H_{0\,\mathrm{K}}^{\mathrm{Ce_2O_3(s)}} = \Delta_{\mathrm{f}} H_{298.15\,\mathrm{K}}^{\mathrm{CeO_2(s)}} - \left(\mathrm{ZPE}^{\mathrm{Ce_2O_3(s)}} - 2\mathrm{ZPE}^{\alpha\text{-Ce(s)}} - \frac{3}{2}\mathrm{ZPE}^{\mathrm{O_2(g)}}\right) = -18.65\,\mathrm{eV}\ . \tag{26}$$

Thus the atomization enthalpies of both oxides are given by

$$\Delta_{\mathrm{at}} H_{0\,\mathrm{K}}^{\mathrm{CeO_2(s)}} = -20.88\,\mathrm{eV}\ , \tag{27}$$

$$\Delta_{\mathrm{at}} H_{0\,\mathrm{K}}^{\mathrm{Ce_2O_3(s)}} = -35.15\,\mathrm{eV}\ , \tag{28}$$

which finally allows us to calculate the experimental zero-temperature reaction enthalpies, corrected by ZPE:

$$\Delta_{r_1} H_{0\,\mathrm{K}} = 3.99\,\mathrm{eV}\ , \tag{29}$$

$$\Delta_{r_2} H_{0\,\mathrm{K}} = 1.26\,\mathrm{eV}\ , \tag{30}$$

$$\Delta_{r_3} H_{0\,\mathrm{K}} = 0.96\,\mathrm{eV}\ . \tag{31}$$

We note that the main uncertainty originates from inconsistent reports of the formation enthalpy of solid Ce_2O_3. Our selected enthalpy corresponds to the careful selection by Konings et al. in Ref. [28]. Taking all their presented formation enthalpies of Ce_2O_3 into account (ranging from -18.9 to $-18.5\,\mathrm{eV}$), results in the following range of zero-temperature reaction enthalpies:

$$\Delta_{r_1} H_{0\,\mathrm{K}} = 3.7\text{–}4.1\,\mathrm{eV}\ , \tag{32}$$

$$\Delta_{r_2} H_{0\,\mathrm{K}} = 1.0\text{–}1.4\,\mathrm{eV}\ , \tag{33}$$

$$\Delta_{r_3} H_{0\,\mathrm{K}} = 0.7\text{–}1.1\,\mathrm{eV}\ . \tag{34}$$

Table XVII: ZPE corrections for all considered compounds. For the bulk the ZPE is per formula unit. Here θ_D denotes the Debye temperature.

Compound	ZPE [eV]	Source
	bulk	
Ce_2O_3	0.18	Debye model ($\theta_D = 373\,\mathrm{K}$) [43]
CeO_2	0.14	Debye model ($\theta_D = 481\,\mathrm{K}$) [43]
α-Ce	0.02	Debye model ($\theta_D = 179\,\mathrm{K}$) [35]
	molecule	
H_2	0.269	coupled cluster calculation [15]
H_2O	0.574	coupled cluster calculation [15]
CO	0.134	coupled cluster calculation [15]
CO_2	0.314	coupled cluster calculation [15]
O_2	0.098	from spectroscopic constants [19]

[1] NIST Computational Chemistry Comparison and Benchmark Database , 2020.
[2] B Amadon, S Biermann, A Georges, and F Aryasetiawan. The α-γ Transition of Cerium Is Entropy Driven. *Phys. Rev. Lett.*, 96(6):066402, 2006.
[3] P. E. Blöchl. Projector augmented-wave method. *Phys. Rev. B*, 50(24):17953–17979, 1994.
[4] Leo Brewer. The cohesive energies of the elements. *The cohesive energies of the elements*, 1977.
[5] Marçal Capdevila-Cortada, Zbigniew Łodziana, and Núria López. Performance of DFT+U approaches in the study of catalytic materials. *ACS Catal.*, 6(12):8370–8379, 2016.

[6] Marco Casadei, Xinguo Ren, Patrick Rinke, Angel Rubio, and Matthias Scheffler. Density functional theory study of the α-γ phase transition in cerium: Role of electron correlation and f -orbital localization. *Phys. Rev. B*, 93(7):075153, 2016.
[7] Juarez L. F. Da Silva, Maria Verónica Ganduglia-Pirovano, Joachim Sauer, Veronika Bayer, and Georg Kresse. Hybrid functionals applied to rare-earth oxides: The example of ceria. *Phys. Rev. B - Condens. Matter Mater. Phys.*, 75(4):19–24, 2007.
[8] L. De'Medici, A. Georges, G. Kotliar, and S. Biermann. Mott transition and kondo screening in f-electron metals. *Phys. Rev. Lett.*, 95(6):5–8, 2005.
[9] Kh E. El-Kelany, C. Ravoux, J. K. Desmarais, P. Cortona, Y. Pan, J. S. Tse, and A. Erba. Spin localization, magnetic ordering, and electronic properties of strongly correlated Ln_2O_3 sesquioxides (Ln=La, Ce, Pr, Nd). *Phys. Rev. B*, 97(24):245118, 2018.
[10] Stefano Fabris, Stefano de Gironcoli, Stefano Baroni, Gianpaolo Vicario, and Gabriele Balducci. Taming multiple valency with density functionals: A case study of defective ceria. *Phys. Rev. B*, 71(4):041102, 2005.
[11] M. Gajdoš, K. Hummer, G. Kresse, J. Furthmüller, and F. Bechstedt. Linear optical properties in the projector-augmented wave methodology. *Phys. Rev. B*, 73(4):045112, 2006.
[12] Shruba Gangopadhyay, Dmitry D. Frolov, Artëm E. Masunov, and Sudipta Seal. Structure and properties of cerium oxides in bulk and nanoparticulate forms. *J. Alloys Compd.*, 584:199–208, 2014.
[13] Antoine Georges, Gabriel Kotliar, Werner Krauth, and Marcelo J. Rozenberg. Dynamical mean-field theory of strongly correlated fermion systems and the limit of infinite dimensions. *Rev. Mod. Phys.*, 68(1):13–125, 1996.
[14] F. Gygi and A. Baldereschi. Self-consistent Hartree-Fock and screened-exchange calculations in solids: Application to silicon. *Phys. Rev. B*, 34(6):4405–4408, 1986.
[15] Trygve Helgaker, Torgeir A. Ruden, Poul Jørgensen, Jeppe Olsen, and Wim Klopper. A priori calculation of molecular properties to chemical accuracy. *J. Phys. Org. Chem.*, 17(11):913–933, 2004.
[16] H. C. Herper, T. Ahmed, J. M. Wills, I. Di Marco, T. Björkman, D. Iuşan, A. V. Balatsky, and Olle Eriksson. Combining electronic structure and many-body theory with large databases: A method for predicting the nature of 4f states in Ce compounds. *Phys. Rev. Mater.*, 1(3):033802, 2017.
[17] Heike C. Herper, Olga Yu Vekilova, Sergei I. Simak, Igor Di Marco, and Olle Eriksson. Localized versus itinerant character of 4f-states in cerium oxides. *J. Phys. Condens. Matter*, 32(21):215502, 2020.
[18] Li Huang and Haiyan Lu. Electronic structure of cerium: A comprehensive first-principles study. *Phys. Rev. B*, 99(4):1–14, 2019.
[19] Karl K. Irikura. Experimental Vibrational Zero-Point Energies: Diatomic Molecules. *J. Phys. Chem. Ref. Data*, 36(2):389–397, 2007.
[20] Hong Jiang and Eberhard Engel. Kohn-Sham perturbation theory: Simple solution to variational instability of second order correlation energy functional. *J. Chem. Phys.*, 125(18):184108, 2006.
[21] Hong Jiang and Eberhard Engel. Random-phase-approximation-based correlation energy functionals: Benchmark results for atoms. *J. Chem. Phys.*, 127(18), 2007.
[22] Hong Jiang, Patrick Rinke, and Matthias Scheffler. Electronic properties of lanthanide oxides from the GW perspective. *Phys. Rev. B*, 86(12):125115, 2012.
[23] B. Johansson, I. A. Abrikosov, M. Aldén, A. V. Ruban, and H. L. Skriver. Calculated phase diagram for the $\gamma \rightleftharpoons \alpha$ transition in ce. *Phys. Rev. Lett.*, 74(12):2335–2338, 1995.
[24] Merzuk Kaltak, Jiří Klimeš, and Georg Kresse. Cubic scaling algorithm for the random phase approximation: Self-interstitials and vacancies in Si. *Phys. Rev. B - Condens. Matter Mater. Phys.*, 90(5):054115, 2014.
[25] Junwon Kim, Dong Choon Ryu, Chang Jong Kang, Kyoo Kim, Hongchul Choi, T. S. Nam, and B. I. Min. Topological phase transition in the archetypal f-electron correlated system of cerium. *Phys. Rev. B*, 100(19), 2019.
[26] Charles Kittel. *Introduction to solid state physics.* John Wiley & Sons, Inc, 8 edition, 2005.
[27] Jiří Klimeš, Merzuk Kaltak, and Georg Kresse. Predictive GW calculations using plane waves and pseudopotentials. *Phys. Rev. B*, 90(7):075125, 2014.
[28] Rudy J. M. Konings, Ondrej Beneš, Attila Kovács, Dario Manara, David Sedmidubský, Lev Gorokhov, Vladimir S. Iorish, Vladimir Yungman, E. Shenyavskaya, and E. Osina. The Thermodynamic Properties of the f-Elements and their Compounds. Part 2. The Lanthanide and Actinide Oxides. *J. Phys. Chem. Ref. Data*, 43(1):013101, 2014.
[29] G. Kresse and J. Furthmüller. Efficient iterative schemes for ab initio total-energy calculations using a plane-wave basis set. *Phys. Rev. B*, 54(16):11169–11186, 1996.
[30] G. Kresse and D. Joubert. From ultrasoft pseudopotentials to the projector augmented-wave method. *Phys. Rev. B*, 59(3):1758–1775, 1999.
[31] Enzhi Li, Shuxiang Yang, Peng Zhang, Ka Ming Tam, Mark Jarrell, and Juana Moreno. Periodic Anderson model with Holstein phonons for the description of the cerium volume collapse. *Phys. Rev. B*, 99(15):155147, 2019.
[32] B. Meredig, A. Thompson, H. A. Hansen, C. Wolverton, and A. van de Walle. Method for locating low-energy solutions within DFT+U, volume = 82, year = 2010. *Phys. Rev. B*, (19):195128.
[33] J. Paier, R. Hirschl, M. Marsman, and G. Kresse. The Perdew–Burke–Ernzerhof exchange-correlation functional applied to the G2-1 test set using a plane-wave basis set. *J. Chem. Phys.*, 122(23):234102, 2005.
[34] Xinguo Ren, Patrick Rinke, Christian Joas, and Matthias Scheffler. Random-phase approximation and its applications in computational chemistry and materials science. *J. Mater. Sci.*, 47(21):7447–7471, 2012.
[35] Joo Yull Rhee. *Optical properties of γ- and α-Ce by spectroscopic ellipsometry.* PhD thesis, Iowa State University, 1992.
[36] Tobias Schäfer. *Low-scaling algorithms for many-body exchange-like diagrams.* PhD thesis, University of Vienna, 2018.
[37] Tobias Schäfer, Benjamin Ramberger, and Georg Kresse. Quartic scaling MP2 for solids: A highly parallelized algorithm

in the plane wave basis. *J. Chem. Phys.*, 146(10):104101, 2017.
[38] Gustavo E. Scuseria, Thomas M. Henderson, and Danny C. Sorensen. The ground state correlation energy of the random phase approximation from a ring coupled cluster doubles approach. *J. Chem. Phys.*, 129(23):231101, 2008.
[39] James Spencer and Ali Alavi. Efficient calculation of the exact exchange energy in periodic systems using a truncated Coulomb potential. *Phys. Rev. B - Condens. Matter Mater. Phys.*, 77(19):1–4, 2008.
[40] Ravishankar Sundararaman and T. A. Arias. Regularization of the Coulomb singularity in exact exchange by Wigner-Seitz truncated interactions: Towards chemical accuracy in nontrivial systems. *Phys. Rev. B*, 87(16):165122, 2013.
[41] Attila Szabo and Neil S Ostlund. *Modern quantum chemistry: introduction to advanced electronic structure theory*. Courier Corporation, 2012.
[42] Ming Feng Tian, Hai Feng Song, Hai Feng Liu, Cong Wang, Zhong Fang, and Xi Dai. Thermodynamics of the α-γ Transition in cerium studied by an LDA + Gutzwiller method. *Phys. Rev. B - Condens. Matter Mater. Phys.*, 91(12):1–5, 2015.
[43] Philippe F Weck and Eunja Kim. Assessing Hubbard-corrected AM05 + U and PBEsol + U density functionals for strongly. *Phys. Chem. Chem. Phys.*, 18(38):26816–26826, 2016.
[44] Zongxian Yang, Tom K. Woo, Micael Baudin, and Kersti Hermansson. Atomic and electronic structure of unreduced and reduced CeO2 surfaces: A first-principles study. *J. Chem. Phys.*, 120(16):7741–7749, 2004.

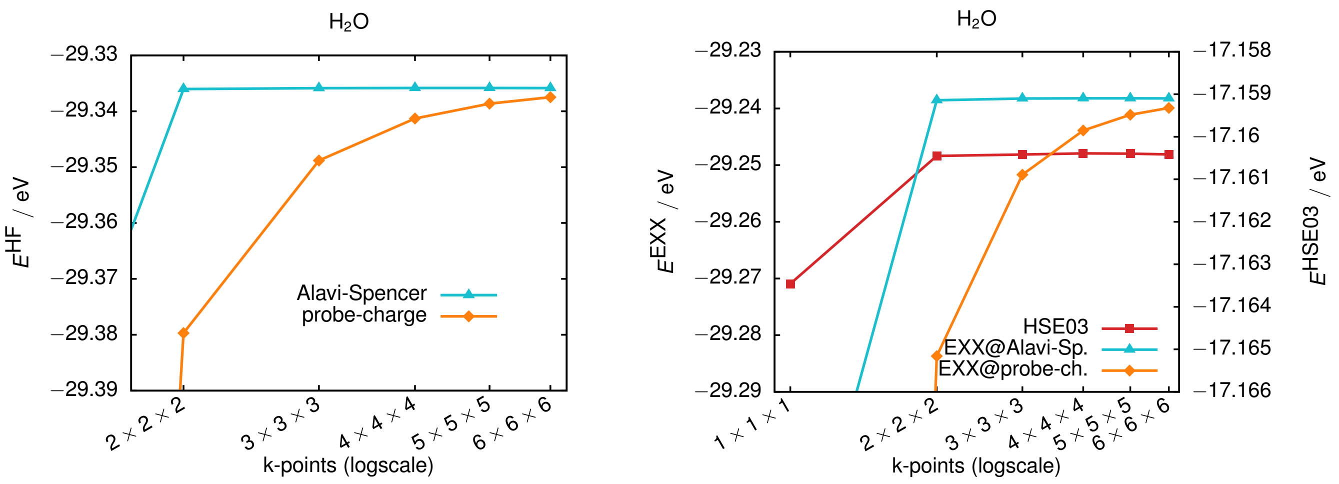

Figure 3: Convergence of the Hartree-Fock energy per molecule of H_2O gas with respect to the employed k-mesh, using the Alavi-Spencer and probe-charge scheme (see section VIII 1). This convergence probes the correction to the unphysical exchange interaction between BvK cells as well as the convergence of the intermolecular electrostatic energy per molecule. We employed a plane-wave cutoff of `ENCUT` $= 600\,\text{eV}$ and a linear unit cell dimension (intermolecular distance) of $L = 6.65\,\text{Å}$.

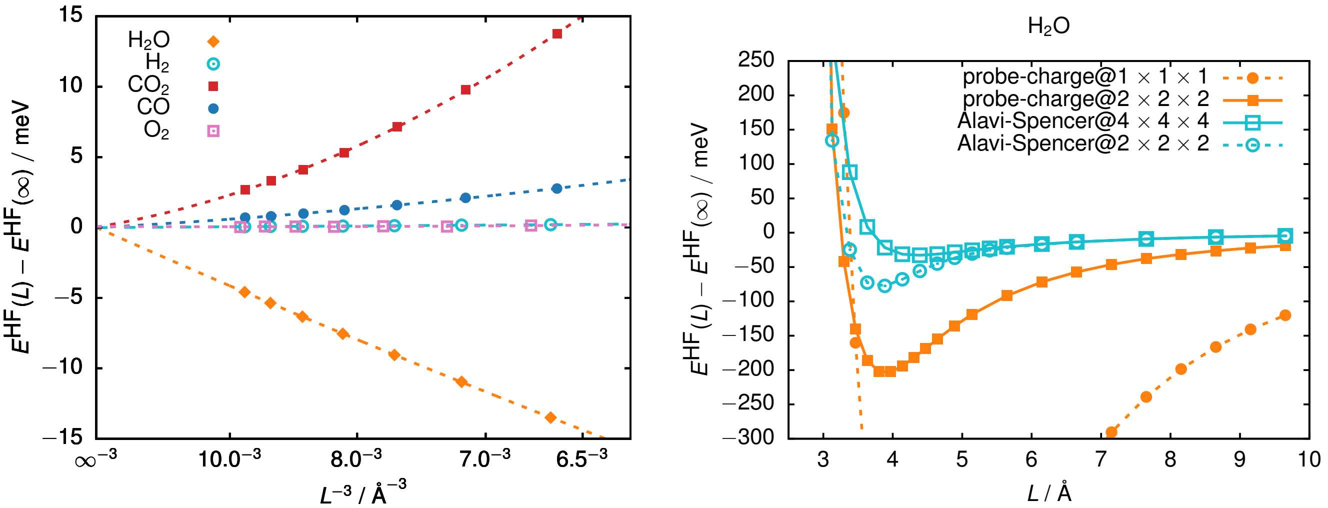

Figure 4: Convergence of the Hartree-Fock energy in the limit of an infinitely diluted gas ($L \to \infty$). To aid the comparison, the energy in the limit is shifted to 0 for all molecules. Left: validity of the fit/extrapolation using the Alavi-Spencer scheme at $2 \times 2 \times 2$ k-points. The dashed lines are fitting functions of the form $E_\infty + \alpha L^{-3} + \beta L^{-6}$. Right: comparison on a broader scale of (i) different k-meshes, and (ii) correction schemes to the spurious exchange interaction, for the case of H_2O. The simplest setting (probe-charge@$1 \times 1 \times 1$) exhibits an excessively attractive behavior. It apparently converges to the same limit as the reference setting (Alavi-Spencer@$4 \times 4 \times 4$), though much slower, so that a high accuracy cannot be expected from an extrapolation. We employed a plane-wave cutoff of `ENCUT` $= 800\,\text{eV}$.

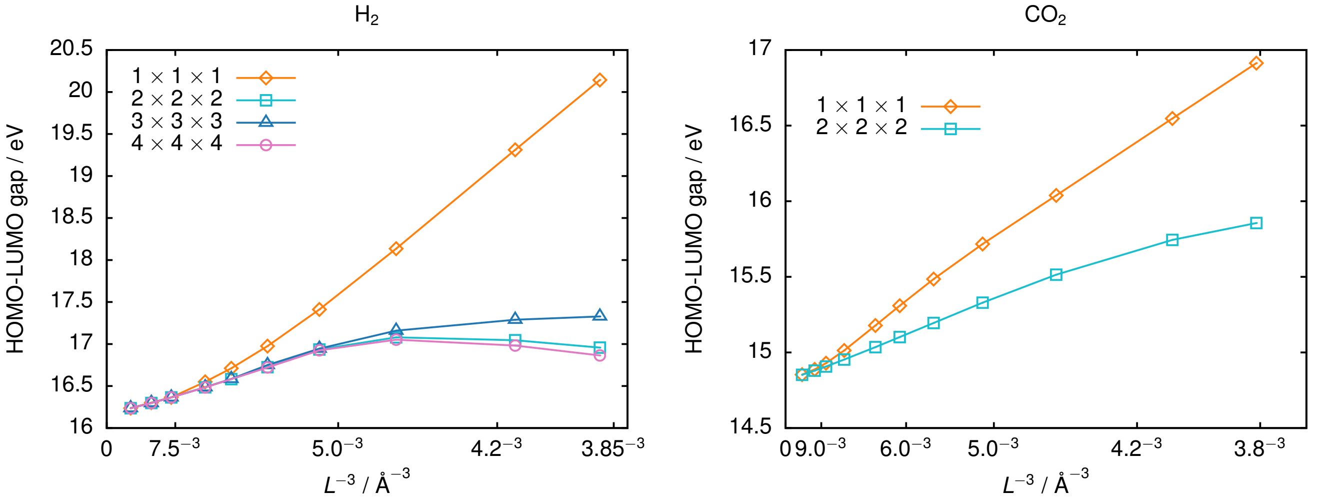

Figure 5: Hartree-Fock HOMO-LUMO gaps as a function of L and the k-mesh. It is apparent that the gap largely closes with L^{-3}.

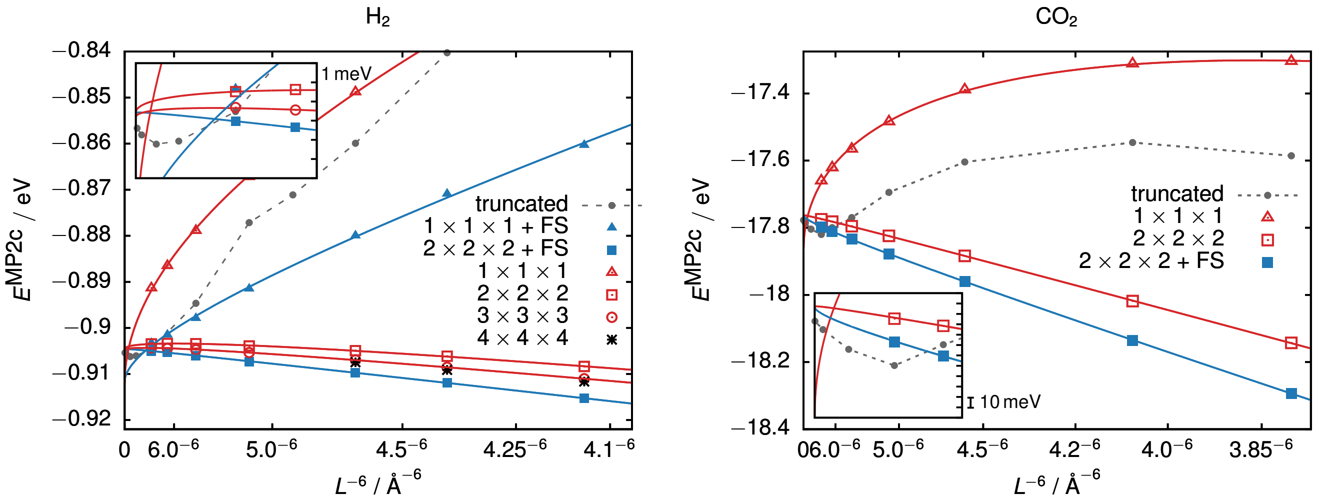

Figure 6: Convergence of the MP2 correlation energy per molecule of gaseous H_2 (left) or CO_2 (right) with respect to the linear unit cell size L (molecular distance), at different k-meshes and finite size corrections (FS). The solid lines are the fitted functions of the form in Eq. (13). We employed a plane-wave cutoff of `ENCUT` $= 600\,$eV.

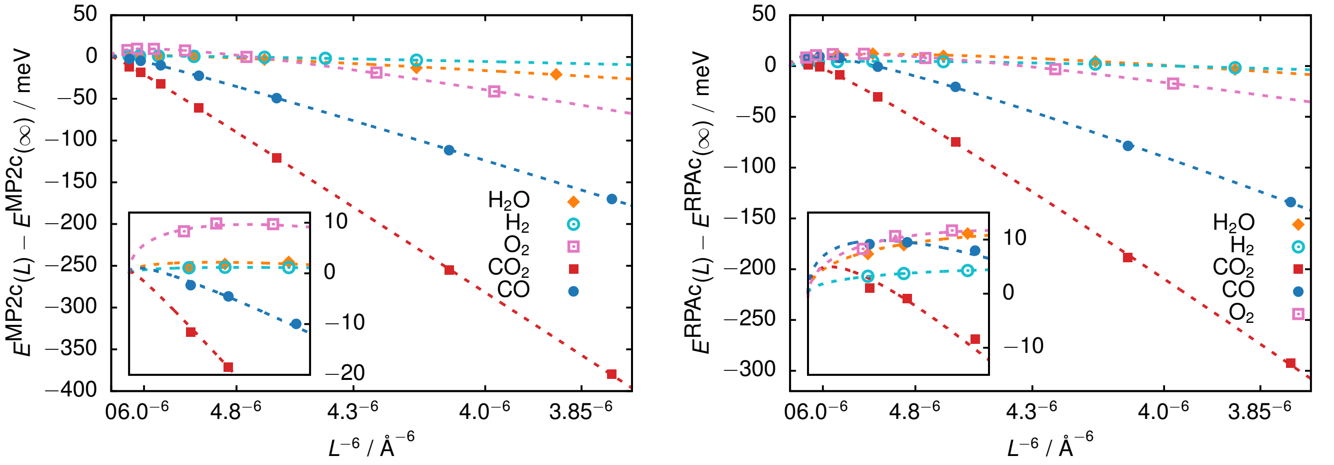

Figure 7: Validity of the fit/extrapolation of the correlation energy to an infinitely diluted gas. To aid the comparison, the energy in the limit is shifted to 0 for all molecules. Left: MP2 extrapolation using $2 \times 2 \times 2$ k-points and `ENCUT` $= 600$. Right: RPA extrapolation using $2 \times 2 \times 2$ k-points and `ENCUT` $= 800$. The dashed lines are fitting functions according Eq. (13).
